# XGBoost based machine learning approach to predict the risk of fall in older adults using gait outcomes

**DOI:** 10.1038/s41598-021-91797-w

**Published:** 2021-06-09

**Authors:** Byungjoo Noh, Changhong Youm, Eunkyoung Goh, Myeounggon Lee, Hwayoung Park, Hyojeong Jeon, Oh Yoen Kim

**Affiliations:** 1 Department of Kinesiology, Jeju National University, Jeju, Republic of Korea; 2 Department of Health Sciences, The Graduate School of Dong-A University, Busan, Republic of Korea; 3 Human Life Research Center, Dong-A University, Busan, Republic of Korea; 4 Department of Child Studies, Dong-A University, Busan, Republic of Korea; 5 Department of Food Science and Nutrition, Dong-A University, Busan, Republic of Korea

**Keywords:** Biomarkers, Health care, Risk factors

## Abstract

This study aimed to identify the optimal features of gait parameters to predict the fall risk level in older adults. The study included 746 older adults (age: 63–89 years). Gait tests (20 m walkway) included speed modification (slower, preferred, and faster-walking) while wearing the inertial measurement unit sensors embedded in the shoe-type data loggers on both outsoles. A metric was defined to classify the fall risks, determined based on a set of questions determining the history of falls and fear of falls. The extreme gradient boosting (XGBoost) model was built from gait features to predict the factor affecting the risk of falls. Moreover, the definition of the fall levels was classified into high- and low-risk groups. At all speeds, three gait features were identified with the XGBoost (stride length, walking speed, and stance phase) that accurately classified the fall risk levels. The model accuracy in classifying fall risk levels ranged between 67–70% with 43–53% sensitivity and 77–84% specificity. Thus, we identified the optimal gait features for accurate fall risk level classification in older adults. The XGBoost model could inspire future works on fall prevention and the fall-risk assessment potential through the gait analysis of older adults.

## Introduction

Falls are among the most common causes of injury, severe health problems, and even death in older adults^1^. Numerous studies have revealed a relationship between falls and risk factors such as advanced age^2^, declined cognitive function^3^, strength deficit, gait abnormalities, and reduced balance^4^. In particular, gait abnormalities in aging, including slow walking speed, greater gait variability, and shorter steps, are considered one of the greatest risk factors for falls^5–8^. Furthermore, gait abnormalities or decreased gait ability decisively imply a reduced physical fitness as a result of aging^9^, which may cause a falls. Thus, the underlying causes of fall must be identified to predict their risk. In addition, it is necessary to identify in advance the influential predictor factors affecting falls through a gait performance test and use them as fundamental data to prevent falls. Furthermore, novel methods are required to overcome the limitations of existing studies.

The machine learning (ML) techniques have gained attention for addressing the clinically relevant spatiotemporal gait parameters for disease classification^10^. The ML techniques use features extracted from a set of clinically relevant data, allowing computer algorithms to form a predictive model^11^. In addition, the ML algorithm can be used to extract the optimal features affecting the risk of falls from the gait features, which measured a more continuative state for longer durations using the inertial measurement unit (IMU) sensors. In this study, we applied the ML technique using the extreme gradient boosting (XGBoost) algorithm^12^, a decision tree-based ensemble ML technique. The XGBoost minimizes the residuals of the models and increases the predictive power by combining weak learners^13^. Using XGBoost, we expect to distinguish the spatiotemporal gait parameters from high and low fall-risk level subjects. To our knowledge, only a few prediction researchers have studied the risk of falls and extracted the essential factors using ML techniques^14,15^. A model using support vector machine with parameter tuning was proposed in^14^. The model was developed to discriminate the balancing problems in older adults; thus, the model does not predict the risk of falling. Moreover, models based on artificial neural networks were developed to examine the efficiency in classifying with or without recurrent falling utilizing a set of clinical characteristics corresponding to risk factors of falls in the older adults^15^. However, the developed models did not consider extracting the essential gait parameters. Furthermore, no studies have used the XGBoost algorithm to classify high and low fall risk levels objectively based on their gait spatiotemporal features.

Spatiotemporal parameters of gait have been used for the classification the falls using ML algorithms^16^. However, previous studies have compared ML models’ performances considering relatively fewer steps in their gait assessment, such as the Timed up and go test. The gait assessment with relatively fewer steps may not yield similar results to an actual walking environment. Research on gait with more continuative states for longer durations strengthens the reliability of spatiotemporal variables using the wearable sensor technology^17^. IMU sensors can measure the gait outside of a laboratory and real-world at low cost than motion capture system with continuative states for longer steps^18^. Thus, gait analysis with numerous consecutive steps is necessary for improving the reliability of gait variables. Additionally, this study was focused on identifying the optimal features of gait parameters to predict the fall risk level in older adults. Therefore, we used the XGBoost algorithm of ML on gait performance tests with speed modification to identify fall risk levels in older adults and define optimal gait parameters.

## Methods

### Participants

Participants were recruited from a community-wide survey in Busan Metropolitan City. Participants satisfied the following exclusion criteria: (1) they were unable to walk without any support, and (2) they have a history of musculoskeletal injuries or neurophysiological problems in the past six months. In total, 746 older adults with ages ranging from 63 to 89 years participated in the study. All methods were performed in accordance with the relevant guidelines and regulations. All participants signed their informed consent after reading all the study details. This study was approved by the Institutional Review Board of Dong-A University (IRB number: 2–104709–AB–N–01–201808–HR–023–02).

### Instrumentation

Gait performance data were collected as previously described by Noh et al.^18^ and Lee et al.^19^. Gait performance tests were evaluated using a gait analysis system (DynaStab, JEIOS, Busan, South Korea), including shoe-type data loggers (Smart Balance SB-1, JEIOS, Busan, South Korea) and embedded IMU sensors (IMU-3000, InvenSense, San Jose, CA, USA) on both outsoles. Gait performance data was collected by triaxial accelerations with up to ± 6 g and triaxial angular velocities with up to ± 500°s^–1^ along three orthogonal axes. Gait performance data were collected at a sampling frequency of 100 Hz using a data acquisition system (Smart Balance version 1.5, JEIOS, Busan, South Korea). Various sizes of shoe-type data loggers were available for each participant. The international physical activity questionnaire-short form was used to estimate their habitual physical activity (PA) levels with respect to the metabolic equivalents (METs/week)^20^.

### Assessment of fall level

**Figure 1:**
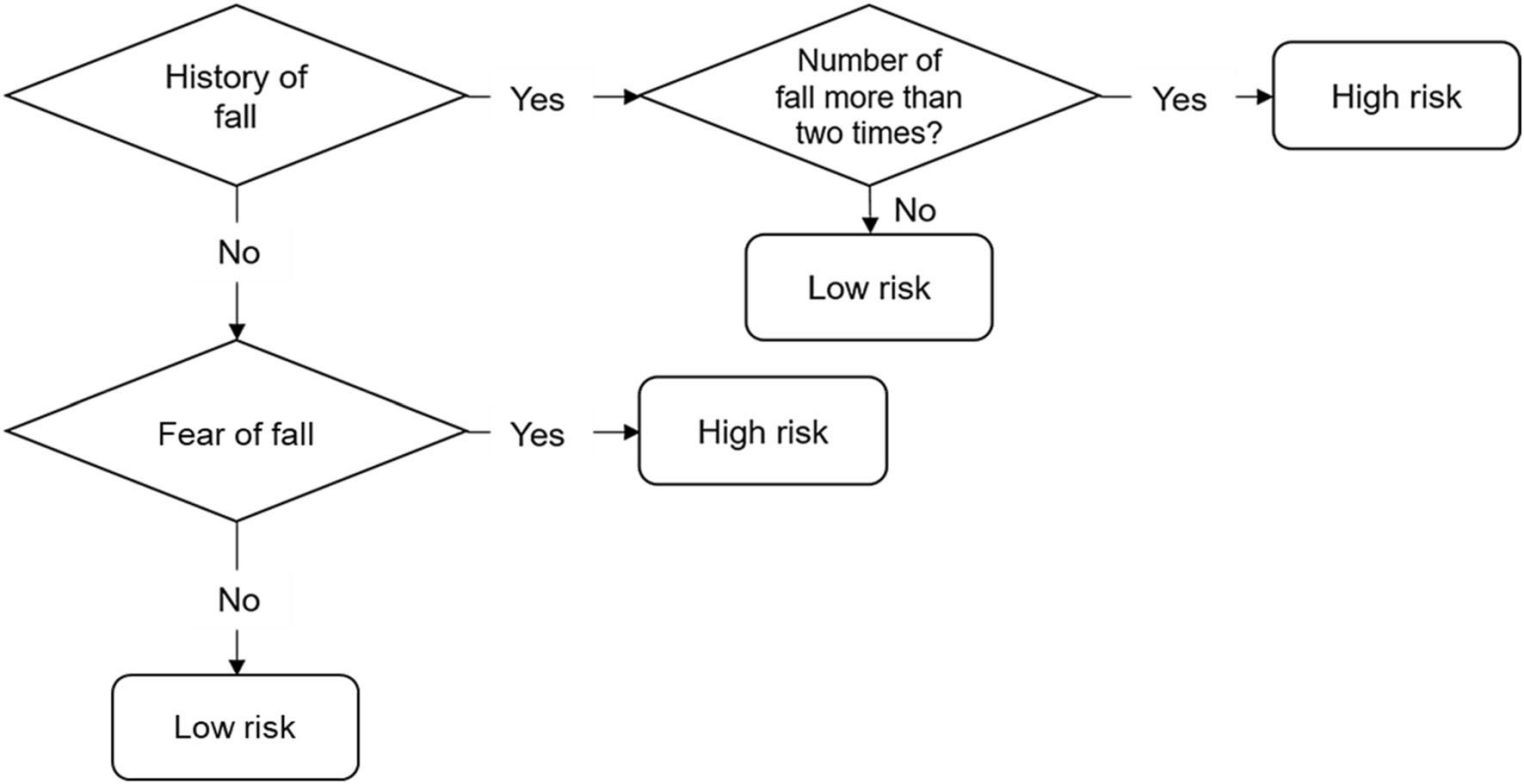
Fall levels definition. This metric was defined to classify the high and low fall risk based on the history of falls and fear of falls questionnaire^21^.

The fall was defined as “an unexpected event in which the person comes to rest on the ground, floor, or lower level.”^21^. Silva et al.^22^ defined a metric to classify the fall risks. In this regard, participants were asked questions concerning their history of falls (Q. Have you fallen in the last 6 months?), number of falls (Q. How many times did you fall in the last 6 months?), and fear of falls (Q. Are you afraid of falling?). Subsequently, a fall risk level was classified along with a metric of fall levels definition, which indicates if the person shows more or less probability to fall (Fig. 1). The low-risk group represented 62% of the dataset, composed of 456 participants. The high-risk group represented approximately 38% of the dataset, including the remaining 290 participants. This distribution follows a similar agreement with the fall incidence in older adults, which is less than 30%^23^.

### Assessment of gait performance

Three gait performance tests were performed on a straight 20-m overground walkway with gait speed modification (slower (– 20% of preferred), self-preferred, and faster (+ 20% of preferred) speeds)^24^. Before the gait performance test, the preferred walking speed was defined using a metronome (beats/min). Participants were asked to perform the walking as close as possible to the targeted slower and faster-walking speed by a metronome. Verbal or visual instructions were provided to perform overground walking. They practiced walking at three-speed conditions using the metronome as a familiarization session for approximately 10 min.

### Data analysis

The gait data were filtered using a second-order Butterworth low-pass filter with a cutoff frequency of 10 Hz^19,25^. Heel strikes and toe-off of gait events were detected when the linear acceleration along the anteroposterior axis and the vertical axis reached its maximum value, respectively^19,25^. We excluded the acceleration and deceleration step periods of the gait performance test to analyze in the steady-state condition. We calculated the spatiotemporal parameters [i.e., walking speed, stride length, cadence, stance phase, stride time, and gait asymmetry (GA)]. The walking speed was calculated using the formula: walking speed = walking distance (m)/walking duration (s). In addition, the cadence, stride time, and stride length were calculated using the formula^26^: The *n* is a total number of heel strike (HS) events.

1 $$Cadence \left( {steps/min} \right){ = }\frac{Step\; counted \times 60}{{Walking\; duration}}$$

2 $$Stride\; time \left( s \right){ = }\frac{1}{n - 1}\mathop \sum \limits_{n = 1}^{n - 1} \frac{{HS_{n + 1} - HS_{n} }}{100}$$

3 $$Stride\; length \left( m \right) { = }\frac{1}{n - 1}\mathop \sum \limits_{n = 1}^{n - 1} (HS_{n + 1} - HS_{n} ) \times walking \;speed$$

The stance phase was also calculated according to the two kinds of formulas^26^. If the *TO*_*n*_ > *HS*_*n*_, then the stance phase was calculated as follow:

4 $$Stance \;phase \left( \% \right)_{case1} { = }\frac{1}{n - 1}\mathop \sum \limits_{n = 1}^{n - 1} \left( {\frac{{TO_{n} - HS_{n} }}{{HS_{n + 1} - HS_{n} }} \times 100} \right)$$

The *n* is the total number of toe off (TO) events. For instance, if the total number of TO is lower than HS events (e.g., 14 vs. 15), the *n* is a total number of TO events (*n* = 14), whereas if the total number of TO is equal to the HS events (e.g., 14), then the *n* is a total number of HS or TO events (*n* = 14). Alternatively, if the *TO*_*n*_ > *HS*_*n*_, then the stance phase was calculated as follow:

5 $$Stance \;phase \left( \% \right)_{case2} { = }\frac{1}{n - 1}\mathop \sum \limits_{n = 1}^{n - 1} \left( {\frac{{TO_{n + 1} - HS_{n} }}{{HS_{n + 1} - HS_{n} }} \times 100} \right)$$

If the total number of TO is greater than HS events (e.g., 13 vs. 12), the *n* is a total number of HS events (*n* = 12), whereas if the total number of TO is equal to the HS events (e.g., 12), then the *n* is a [total number of HS or TO events – 1, *n* = 11]. Walking speed and stride length were normalized by the height of each participant. Moreover, the variability of the stride length, stride time, and stance phase were quantified as the coefficient of variance (CV; standard deviation/mean × 100). The GA was measured according to the bilateral differences between the left and the right limbs during walking^27^, and the formula is as follow:

6 $$ {\text{Gait asymmetry (\%) = }}\left| {{\text{ln}}\left( {\frac{Short \;swing\; time}{{Long\; swing\; time}}} \right)} \right| \times 100$$

The swing time was calculated for each left and right side, and the Long swing time is defined greater mean value between the left and right sides, whereas the short swing time is a relatively lower mean value^27^. The proposed ML model considered five demographic variables (age, sex, body mass index (BMI), total PA, and education levels) and nine gait variables (walking speed, stride length, cadence, stance phase, stride time, CV of stride length, CV of stance phase, CV of stride time, GA) as predictor variables.

### Statistical analysis

The gait data was evaluated through the Shapiro–Wilk test for normality. The gait variables were normalized into max–min scores for all variables. An independent sample *t*-test was used to determine significant differences in characteristics between all participants (high-risk and low-risk group) and demographics.

To predict the factors affecting the risk of falls by the spatiotemporal parameters of gait at three different speeds, we derived the ML technique using the XGBoost algorithm. The ML technique aims to find a relationship between the input *X* = {*x*_*1*_*, x*_*2*_*, …, x*_*N*_} and the output *Y*. As described above, relying on the fall-levels definition, the risk of falls was classified into high- and low-risk groups.

For a given dataset with *n* samples and *m* features, *K* additive functions are used in the XGBoost model to predict the output through the following estimation^12^:

7 $$\hat{y}_{j } { = }\mathop \sum \limits_{k = 1}^{K} f_{k} \left( {x_{i} } \right),$$

where ${f}_{k}\in \left\{f(x)={\omega }_{q}\right\}\left(q : {\mathbb{R}}^{m}\to T, \omega \in {\mathbb{R}}^{T}\right)$ is the regression tree’s space, and *q* denotes the independent structure of each tree with *T* leaves. Each *f*_*k*_ corresponds to an independent tree structure *q* and leaf weights *ω*. To learn the set of functions, the following regularized objective is minimized.

8 $${\mathcal{L}}{ } = { }\mathop \sum \limits_{i} l(\hat{y}_{i} , y_{i} ) + \mathop \sum \limits_{k} \Omega \left( {f_{k} } \right),$$

where $\Omega \left(f\right)= \gamma T+ \frac{1}{2}\uplambda {\Vert \omega \Vert }^{2}$, *l* denotes the model loss function, and Ω denotes the regularized term. The dataset was split into a training set (70%) and a testing set (30%). Ten-fold cross-validation with a random split was used for all the processes.

The model was measured using the prediction performance of the model by computing C-statistics (i.e., the area under the receiver operating characteristic [ROC] curve), prospective prediction results, and decision curves. The accuracy of each identified parameter was estimated as follows:

9 $$Accuracy = \frac{{\left( {True \;Positive + True \;Negative} \right)}}{{\left( {True \;Positive + False \;Negative + False \;Positive + True \;Negative} \right)}}$$

The sensitivity, measuring how accurately the high-risk fall group is identified, and the specificity, measuring how accurately the low-risk fall group is identified, were calculated as

10 $$Sensitivity{ } = { }\frac{True \;Positive}{{\left( {True \;Positive + False \;Negative} \right)}}$$

11 $$Specificity = \frac{True \;Negative}{{\left( {True \;Negative + False\; Positive} \right)}}$$

Using the area under the ROC curves, we evaluated the accuracy of the gait variables in predicting the risk of falls in older adults. All models were adjusted by age, sex, BMI, level of education, and PA levels as covariates. Level of education was defined as a categorical variable (elementary school education or less, middle school education, high school education, college degree, or higher). All analyses were performed with R statistical software (version 3.6.1, RStudio). The level of statistical significance was set at 0.05.

## Results

Table 1 shows the demographic and cognitive characteristics of participants. Compared to the participant in the low-risk fall group, participants with a high risk of falls were relatively older, with higher BMI, lower PA levels and education levels, and poorer cognition. From Table 2, 23 of 27 gait variables were significantly impaired in the high-risk of the fall group. The gait variables presented highly correlated characteristics, which is essential, as these gait variables are not independent regarding their correlation.

**Table 1:** Demographic characteristics of participants.

Variables	All participants (n = 746)	High-risk (n = 290)	Low-risk (n = 456)	*p*-value
Age (years)	73.0 ± 5.2	74.2 ± 5.4	72.2 ± 4.9	**< 0.001**
Height (cm)	157.4 ± 8.1	158.0 ± 8.2	156.9 ± 8.1	0.070
Body weight (kg)	61.3 ± 8.8	61.4 ± 8.9	61.2 ± 8.7	0.729
Body mass index (kg/m^2^)	24.7 ± 2.9	25.1 ± 3.1	24.5 ± 2.7	**0.003**
Total PA (MET-min/week)	1874.3 ± 1789.1	1389.7 ± 1409.7	2182.6 ± 1932.4	**< 0.001**
Education (levels)	2.1 ± 1.1	1.7 ± 1.1	2.3 ± 1.1	**< 0.001**
MMSE score	26.5 ± 2.9	25.7 ± 3.3	26.9 ± 2.6	**< 0.001**

*Mean ± SD* mean and standard deviation, *PA* physical activity, *METs* metabolic equivalents, *MMSE* mini-mental state examination; the level of education was defined as a categorical variable [elementary school education or less (education level 1), middle school education (education level 2), high school education (education level 3), college degree (education level 4), or higher (education level 5)]; boldface denotes a significant difference between high- and low-risk of falls; significant difference: *p* < 0.05.

**Table 2:** Gait characteristics of participants.

Variables	All participants (n = 746)	High-risk (n = 290)	Low-risk (n = 456)	*p*-value
**Slower speed**				
Walking speed (m/s)	0.9 ± 0.1	0.8 ± 0.1	0.9 ± 0.1	< 0.001
Stride length (m)	1.1 ± 0.1	1.1 ± 0.1	1.2 ± 0.1	< 0.001
Cadence (beats/min)	97.8 ± 10.9	97.9 ± 11.1	97.7 ± 10.7	0.799
Stance phase (%)	59.2 ± 1.6	59.5 ± 1.7	59.0 ± 1.6	< 0.001
Stride time (s)	1.2 ± 0.1	1.2 ± 0.1	1.2 ± 0.1	0.895
CV of stride length (%)	2.7 ± 1.3	3.0 ± 1.4	2.5 ± 1.2	< 0.001
CV of stance phase (%)	4.4 ± 2.2	5.0 ± 2.5	4.2 ± 2.0	< 0.001
CV of stride time (%)	2.7 ± 1.3	3.0 ± 1.4	2.5 ± 1.1	< 0.001
Gait asymmetry (%)	2.6 ± 2.4	2.8 ± 2.6	2.5 ± 2.3	0.123
**Preferred speed**				
Walking speed (m/s)	1.2 ± 0.2	1.1 ± 0.2	1.2 ± 0.2	< 0.001
Stride length (m)	1.2 ± 0.2	1.1 ± 0.2	1.3 ± 0.1	< 0.001
Cadence (beats/min)	115.9 ± 10.2	114.8 ± 10.3	116.7 ± 10.1	0.016
Stance phase (%)	57.5 ± 1.7	58.0 ± 1.8	57.2 ± 1.6	< 0.001
Stride time (s)	1.0 ± 0.1	1.0 ± 0.1	1.0 ± 0.1	0.014
CV of stride length (%)	2.0 ± 1.1	2.2 ± 1.3	1.9 ± 0.9	< 0.001
CV of stance phase (%)	3.0 ± 1.7	3.3 ± 2.0	2.8 ± 1.4	0.001
CV of stride time (%)	2.0 ± 1.1	2.2 ± 1.3	1.9 ± 0.9	< 0.001
Gait asymmetry (%)	2.0 ± 2.0	2.1 ± 2.1	2.0 ± 2.0	0.752
**Faster speed**				
Walking speed (m/s)	1.5 ± 0.2	1.4 ± 0.2	1.5 ± 0.2	< 0.001
Stride length (m)	1.4 ± 0.2	1.3 ± 0.2	1.4 ± 0.2	< 0.001
Cadence (beats/min)	130.3 ± 11.7	127.8 ± 12.1	131.8 ± 11.1	< 0.001
Stance phase (%)	55.7 ± 1.8	56.3 ± 1.9	55.3 ± 1.7	< 0.001
Stride time (s)	0.9 ± 0.1	0.9 ± 0.1	0.9 ± 0.1	< 0.001
CV of stride length (%)	1.9 ± 1.0	2.0 ± 1.3	1.7 ± 0.8	0.001
CV of stance phase (%)	2.4 ± 1.3	2.6 ± 1.4	2.2 ± 1.1	< 0.001
CV of stride time (%)	1.9 ± 1.0	2.0 ± 1.3	1.7 ± 0.8	< 0.001
Gait asymmetry (%)	2.1 ± 2.0	2.3 ± 2.2	1.9 ± 1.8	0.015

*Mean ± SD* mean and standard deviation, *CV* coefficient of variance, a significant difference between high- and low-risk of falls: *p* < 0.05.

### Selected optimal features of gait by XGBoost

**Figure 2:**
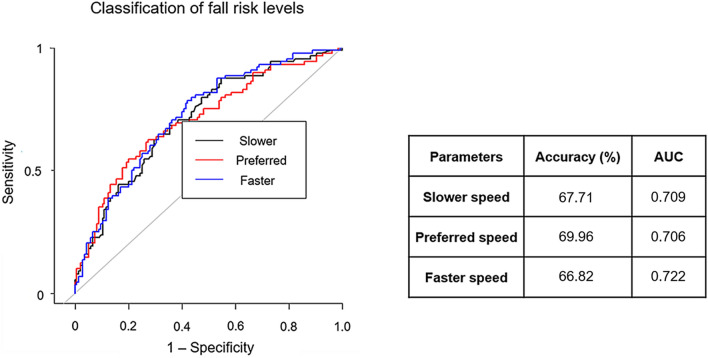
Prediction ability of the XGBoost models for three different walking speeds. ROC curves show the superiority in classifying high vs low level of fall risk. The corresponding values of AUC for each speed are presented.

The XGBoost algorithm was used to extract the optimal features affecting the risk of falls from a total of 34 features. The classification model considered high- and low-risk groups according to fall risk levels. Figure 2 shows the ROC curves; the corresponding values of area under the curve (AUC) for each speed are presented, and the accuracy of each classification model was approximately 68%, 70%, and 67% in the slower-walking, preferred-walking, and faster-walking speed models, respectively. Moreover, the sensitivities were approximately 43%, 53%, and 51% in the slower-walking, preferred-walking, and faster-walking speed models, respectively. The specificities were approximately 84%, 81%, and 77% in the slower walking, preferred walking, and faster-walking speed models, respectively (Table 3).

**Table 3:** Prediction results of the three different walking speed models of the XGBoost in the risk of falls.

Models	AUC (95% CI)	Sensitivity (95% CI)	Specificity (95% CI)	PPV (95% CI)	NPV (95% CI)	PLR (95% CI)	NLR (95% CI)
Slower speed	0.71 (0.64–0.78)	0.43 (0.33–0.54)	0.84 (0.76–0.89)	0.63 (0.50–0.75)	0.69 (0.62–0.76)	2.65 (1.69–4.16)	0.68 (0.56–0.83)
Preferred speed	0.71 (0.64–0.78)	0.53 (0.42–0.64)	0.81 (0.73–0.87)	0.64 (0.52–0.75)	0.73 (0.65–0.80)	2.77 (1.87–4.12)	0.58 (0.45–0.73)
Faster speed	0.72 (0.66–0.79)	0.51 (0.40–0.62)	0.77 (0.69–0.84)	0.59 (0.47–0.70)	0.71 (0.63–0.78)	2.23 (1.54–3.23)	0.63 (0.53–0.83)

*AUC*area under the curve, *NLR* negative likelihood ratio, *NPV* negative predictive value, *PLR* positive likelihood ratio, *PPV* positive predictive value, *CI* confidence interval.

**Figure 3:**
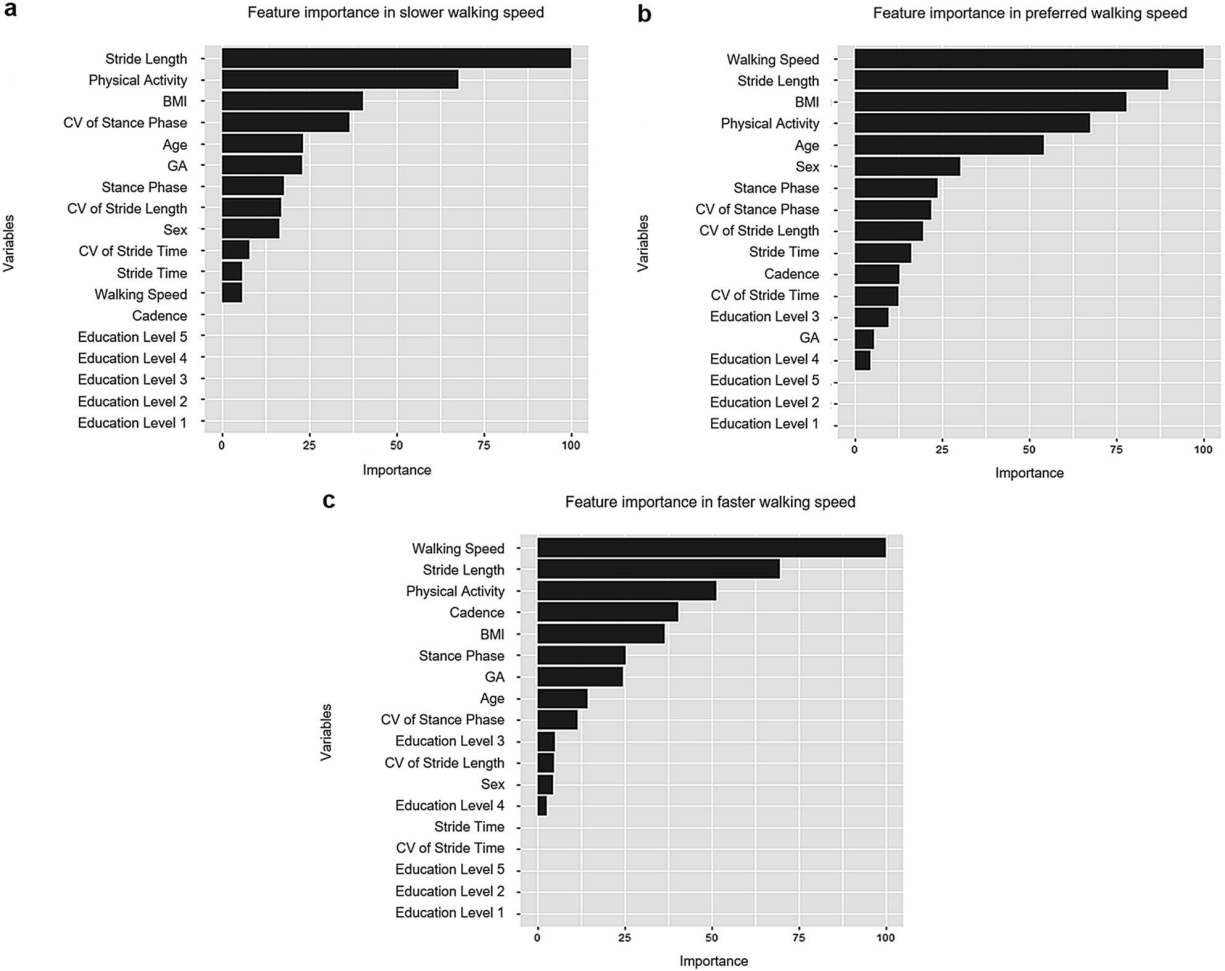
Important features for each walking speeds using the XGBoost models. (**a**) Results of feature selection for the slower-walking, (**b**) preferred-walking, and (**c**) faster-walking speed models.

In the study, the feature importance was calculated through XGBoost to determine the features having an optimal effect when determining the risk of falls. The feature importance is the score result indicating how each variable contributes to the model accuracy when creating the XGBoost model. Figure 3 shows the result of deriving the importance of the main features among all the explanatory variables. As shown in Fig. 3, the most important features are stride length (slower speed) and walking speed (preferred and faster speed) for determining in which the fall into a high- or low-risk groups. Additional important features in the slower-walking speed model among the top 10 were CV of stance phase, GA, stance phase, CV of stride length, CV of stride time, and non-spatiotemporal parameters such as PA level, BMI, age, and gender. Additional features in the preferred-walking speed model included stride length, stance phase, CV of stance phase and stride length, and stride time with non-spatiotemporal parameters such as BMI, PA level, age, and gender among the top 10. Additional features in the faster-walking speed model among the top 10 included the stride length, cadence, stance phase, GA, and CV of stance phase with non-spatiotemporal parameters such as PA level, BMI, and age. Overall, the stride length and stance phase were the common features among the top 10 in all walking speeds models. The variability domain appeared to be an important factor in the risk of falls in older adults when the walking speed was slow. Moreover, as the walking speed increased, the pace and rhythm domains appeared to be important factors.

## Discussion

The analysis of 746 data from older adults was performed using the ML algorithm XGBoost, an approach that allows the identification of optimal features of gait to predict the risk of falls. The XGBoost model achieved high predictive performance using only gait variables. The developed model also achieved acceptable sensitivity and specificity for predicting the risk of falls. To our best knowledge, this is the first study that has applied the ML approach using the XGBoost to identify the predicting gait features for the risk of fall analysis in older adults. The main findings of this study can be summarized as follows: (1) Stride length, walking speed, and stance phase of gait features were identified using XGBoost; these features accurately classified the fall risk levels. (2) The most relevant features were preferred- and faster-walking speed to determine in which group, high- or low-risk, falls can be classified. (3) The XGBoost algorithm could be a useful tool to identify the predicting gait features of the risk of falls in older adults. These findings are discussed in detail below.

Nine gait variables in each walking speed were used as input features to identify gait variables for predicting the risk of falls. In our model, the stride length at slower-walking and the walking speed variable at preferred-walking and faster-walking speeds were the most important features to predict the risk of falls. Moreover, the stance phase is also the common variable among the top 10 for all walking speeds models. In gait assessment, gait variables such as the walking speed have been associated with a high risk of falls^6,28^. A previous study found that a decline in walking speed is one of the early markers of falls^29^. The age-related gait characteristics change in older adults with slow walking speed and a shorter stride (or step) length, could lead to an increase in the stance phase. Thus, walking speed could not always be considered as an independent variable to predict the falls. Simultaneously, the stride length and stance phase were also important features to predict the risk of falls, as shown in our models; therefore, these gait variables should be considered together. The slow walking speed with a shorter stride length may contribute to a longer stance phase in response to the insufficient generating capacity of lower extremity torque. This could result from the force–length relationship, owing to lower strength in older adults, because walking speed is modulated using propulsive force generation during the stance phase of walking^18,30^. This gait pattern may produce dynamic instability, which could lead to an increase in the risk of falls^7^. Moreover, a longer stance phase disrupted the gait harmony^31,32^ (golden ratio between the stance and the swing phase) caused by an impairment in the reciprocal circuits between the cerebellum and the basal ganglia. This can be involved in the regulation of gait, because the overlapped area cooperates to modulate the motor and cognitive functions during walking in the older adults^33,34^. This is supported by our previous studies where a longer stance phase, owing to the slow walking speed with a shorter stride length, as well as decreased muscular strength, was strongly associated with the lower global cognitive functions in older adults^18,30^. The results also showed that global cognitive function in a high-risk group indicated lower cognitive functions than a low-risk group. Moreover, our findings are similar to a previous study where the association between an increase in gait variability and an increase in fall risks in older adults was analyzed^35^. Our result showed that the variability domain appeared to be an important factor in the high risk of falls when the walking speed was slow. Gait variability is increased in response to the stride-to-stride fluctuations to generate force using muscle in the aging process with the partial summation of overlapping twitches due to impaired cognitive functions during modulation in slow walking^18^.

Our results showed that one of the most important features was preferred and faster walking speed to determine in which group, high- or low-risk, the fall can be classified. Namely, an increase in walking speed may increase the risk of falls rather than the slow walking speed. Walking can be defined as a process of continuous loss and recovery of balance that initiates as the center of mass (COM) moving forward, translating the body system mechanically, and recovering a dynamic balance by moving another foot forward to avoid falls^36^. The COM motion in the mediolateral direction could decrease, whereas in the vertical direction could increase as the walking speed increases following a sinusoidal pattern^37^. Thus, altering the COM motion due to the increase in walking speed may contribute to the decline of dynamic stability during walking^38^. Furthermore, dynamic instability due to impaired postural regulation during walking in older adults increases the potential risk of falls because the postural regulation may be integrated through the descending commands for movement being transmitted to brainstem, which is involved in postural control, providing a way to adjust the magnitude and timing of postural changes during stance phase^39^. On increasing the walking speed, this impaired postural regulation could not dissipate the momentum generated with a fast walking speed despite the momentum control of COM being essential to maintain the dynamic stability^38^.

Selection of the ML techniques to predict the factor affecting the risk of falls was based on ML framework. Different ML models such as XGBoost, logistic regression, classification and regression tree, random forest, and deep learning were employed (Tables S1–S3). However, in this study, better fall status prediction results were obtained using the XGBoost. Based on our three different walking speed models, we suggest that the XGBoost algorithm could be a useful tool to identify the predicting gait features of the risk of falls in older adults. In the models, the results can classify the high-risk group from the low-risk group with an overall accuracy ranging from 67 to 70% with the sensitivity ranging from 43 to 53% and specificity ranging between 77 and 84%. A previous study reported that the XGBoost algorithm showed high predictive classification accuracy on falls, which is similar to our models^40^. In addition, the preferred walking speed model had better classification ability to predict the risk of falls among three different walking speed models. The selected optimal features of gait obtained by XGBoost are similar to numerous previous studies regarding the features predicting the risk of falls^5–8^. Therefore, these findings pave the way for a better understanding of the utility of ML-XGBoost algorithm to help informed prediction of potential falls.

Our study presented several potential limitations. First, we were unable to consider the fall efficacy scale, assessing the fear of falls. We evaluated the fear of falls using only the question that ‘Are you afraid of falls? However, we assumed that our fall-levels definition could be properly classified as the risk of falls, even though only one question was asked to assess fear of falls. Second, our datasets have an imbalance between sex and age. Older adults are reported as a risk factor of falls^2,41^. The classification results should improve with a more homogeneous dataset. Lastly, one may assume that the relatively higher distribution datasets of high-risk groups might affect the predictability (lower sensitivities). To improve the classification performance, a comparison of the ML models’ performances should be conducted. Moreover, ML techniques with higher predictability and a filtering technique for human motion should be developed. A method considering the further expanding of the number of samples or collecting various samples and additional variables contributing to improving the predictability can be added to the classification model. These additional possible ways could improve the XGBoost model classification or even other transparent models. However, we concluded that the three XGBoost approaches consistently showed outstanding predictability. Further studies should evaluate the findings on a much larger dataset in realistic environmental conditions.

## Conclusions

In this study, the ML-XGBoost approach was used to identify the most important features for predicting the risk of falls in older adults. The XGBoost algorithm showed the highest classification accuracy of 70% and selected the optimal features such as stride length at slower-walking and the walking speed variable at preferred-walking and faster-walking speeds. Moreover, the stance phase in all walking speeds was also selected as the optimal feature for precisely fall risk levels classification in older adults. Additionally, the results showed that the increase in walking speed should increase fall risks. These gait features should be considered for predicting the risk of falls in older adults. The fall risk assessment by the ML approaches with inertial measurement unit sensors improved the classification of the individuals with a high risk of falling. Our results are useful for the foundation for future works on fall prevention. Moreover, our ML approaches could inspire the fall risk potential assessment through the gait analysis of older adults.

## Supplementary Information

Supplementary File 1.

Supplementary Tables.
