# Supplementary material for: XGBoost based machine learning approach to predict the risk of fall in older adults using gait outcomes
Source: Sci Rep. 2021 Jun 9;11:12183. doi: 10.1038/s41598-021-91797-w (PMC8190134; doi:10.1038/s41598-021-91797-w)
Supplement: Supplementary file 2 — Supplementary Tables. [file 41598_2021_91797_MOESM2_ESM.docx]

**Supplementary Table S1.** Prediction results of slower walking speed models of the models in the risk of falls.

| **Models** | **AUC**  **(95% CI)** | **Sensitivity**  **(95% CI)** | **Specificity**  **(95% CI)** | **PPV**  **(95% CI)** | **NPV**  **(95% CI)** | **PLR**  **(95% CI)** | **NLR**  **(95% CI)** |
| --- | --- | --- | --- | --- | --- | --- | --- |
| XGBoost | 0.71  (0.64-0.78) | 0.43  (0.33-0.54) | 0.84  (0.76-0.89) | 0.63  (0.50-0.75) | 0.69  (0.62-0.76) | 2.65  (1.69-4.16) | 0.68  (0.56-0.83) |
| Logistic Regression | 0.71  (0.64-0.78) | 0.45  (0.35-0.56) | 0.84  (0.77-0.90) | 0.66  (0.52-0.77) | 0.70  (0.63-0.77) | 2.92  (1.85-4.60) | 0.65  (0.53-0.79) |
| Classification and Regression Tree | 0.66  (0.60-0.72) | 0.32  (0.22-0.43) | 0.91  (0.85-0.95) | 0.70  (0.53-0.83) | 0.67  (0.60-0.74) | 3.58  (1.92-6.66) | 0.75  (0.64-0.87) |
| Random Forest | 0.70  (0.63-0.78) | 0.32  (0.22-0.43) | 0.87  (0.81-0.92) | 0.62  (0.47-0.76) | 0.66  (0.59-0.73) | 2.53  (1.47-4.33) | 0.78  (0.67-0.91) |
| Deep Learning | 0.64  (0.56-0.71) | 0.43  (0.33-0.54) | 0.70  (0.61-0.77) | 0.48  (0.37-0.60) | 0.65  (0.57-0.73) | 1.42  (1.00-2.02) | 0.82  (0.66-1.01) |

XGBoost, the extreme gradient boosting; AUC, area under the curve; NLR, negative likelihood ratio; NPV, negative predictive value; PLR, positive likelihood ratio; PPV, positive predictive value; CI, confidence interval.

**Supplementary Table S2.** Prediction results of preferred walking speed models of the models in the risk of falls.

| **Models** | **AUC**  **(95% CI)** | **Sensitivity**  **(95% CI)** | **Specificity**  **(95% CI)** | **PPV**  **(95% CI)** | **NPV**  **(95% CI)** | **PLR**  **(95% CI)** | **NLR**  **(95% CI)** |
| --- | --- | --- | --- | --- | --- | --- | --- |
| XGBoost | 0.71  (0.64-0.78) | 0.53  (0.42-0.64) | 0.81  (0.73-0.87) | 0.64  (0.52-0.75) | 0.73  (0.65-0.80) | 2.77  (1.87-4.12) | 0.58  (0.45-0.73) |
| Logistic Regression | 0.72  (0.65-0.79) | 0.50  (0.39-0.61) | 0.84  (0.76-0.89) | 0.67  (0.54-0.78) | 0.72  (0.64-0.79) | 3.07  (1.98-4.74) | 0.60  (0.48-0.75) |
| Classification and Regression Tree | 0.65  (0.59-0.72) | 0.68  (0.57-0.78) | 0.60  (0.51-0.68) | 0.53  (0.43-0.62) | 0.74  (0.65-0.82) | 1.70  (1.33-2.19) | 0.53  (0.38-0.74) |
| Random Forest | 0.73  (0.67-0.80) | 0.44  (0.34-0.55) | 0.87  (0.81-0.92) | 0.70  (0.56-0.81) | 0.71  (0.63-0.77) | 3.52  (2.13-5.82) | 0.64  (0.52-0.78) |
| Deep Learning | 0.67  (0.60-0.74) | 0.74  (0.63-0.83) | 0.53  (0.44-0.61) | 0.50  (0.41-0.59) | 0.76  (0.66-0.84) | 1.56  (1.25-1.94) | 0.50  (0.34-0.73) |

XGBoost, the extreme gradient boosting; AUC, area under the curve; NLR, negative likelihood ratio; NPV, negative predictive value; PLR, positive likelihood ratio; PPV, positive predictive value; CI, confidence interval.

**Supplementary Table S3.** Prediction results of faster walking speed models of the models in the risk of falls.

| **Models** | **AUC**  **(95% CI)** | **Sensitivity**  **(95% CI)** | **Specificity**  **(95% CI)** | **PPV**  **(95% CI)** | **NPV**  **(95% CI)** | **PLR**  **(95% CI)** | **NLR**  **(95% CI)** |
| --- | --- | --- | --- | --- | --- | --- | --- |
| XGBoost | 0.72  (0.66-0.79) | 0.51  (0.40-0.62) | 0.77  (0.69-0.84) | 0.59  (0.47-0.70) | 0.71  (0.63-0.78) | 2.23  (1.54-3.23) | 0.63  (0.50-0.80) |
| Logistic Regression | 0.74  (0.68-0.81) | 0.47  (0.36-0.58) | 0.86  (0.79-0.91) | 0.68  (0.55-0.80) | 0.71  (0.64-0.78) | 3.31  (2.06-5.31) | 0.62  (0.51-0.76) |
| Classification and Regression Tree | 0.67  (0.60-0.74) | 0.53  (0.42-0.64) | 0.77  (0.69-0.84) | 0.60  (0.49-0.71) | 0.72  (0.64-0.79) | 2.33  (1.61-3.35) | 0.60  (0.47-0.77) |
| Random Forest | 0.74  (0.67-0.80) | 0.45  (0.35-0.56) | 0.83  (0.76-0.89) | 0.63  (0.50-0.75) | 0.70  (0.62-0.77) | 2.67  (1.72-4.13) | 0.66  (0.54-0.81) |
| Deep Learning | 0.71  (0.64-0.78) | 0.49  (0.38-0.60) | 0.77  (0.69-0.84) | 0.58  (0.46-0.69) | 0.70  (0.62-0.77) | 2.13  (1.46-3.10) | 0.66  (0.53-0.83) |

XGBoost, the extreme gradient boosting; AUC, area under the curve; NLR, negative likelihood ratio; NPV, negative predictive value; PLR, positive likelihood ratio; PPV, positive predictive value; CI, confidence interval.
